# Training for Awareness, Resilience and Action (TARA) for medical students: a single-arm mixed methods feasibility study to evaluate TARA as an indicated intervention to prevent mental disorders and stress-related symptoms

**DOI:** 10.1186/s12909-022-03122-2

**Published:** 2022-02-28

**Authors:** Erik Ekbäck, Johanna von Knorring, Anna Burström, David Hunhammar, Inga Dennhag, Jenny Molin, Eva Henje

**Affiliations:** 1 Department of Clinical Science, Umeå University, Umeå, Sweden; 2 Department of Nursing, Department of Clinical Science, Umeå university, Umeå, Sweden

**Keywords:** Medical students, Mental health, Depression, Anxiety, Psychological stress, Qualitative research

## Abstract

**Background:**

Medical students have a higher risk for depression, anxiety, stress-related symptoms, burnout, and suicide, and more rarely seek professional help or treatment than the general population. Appeals are being made to address the mental health and resilience of physicians-to-be. The novel program Training for Awareness, Resilience, and Action (TARA) was originally developed to treat depressed adolescents, targeting specific neuroscientific findings in this population. TARA has shown feasibility and preliminary efficacy in clinically depressed adolescents and corresponding brain-changes in mixed community adolescent samples. The present study investigated the feasibility and acceptability of TARA as a potential indicated prevention program for symptoms of depression, anxiety, stress and burnout in Swedish medical students.

**Methods:**

We conducted a single-arm trial with 23 self-selected students in their early semesters of medical school (mean age 25.38 years, 5 males and 18 females), with or without mental disorders. All participants received TARA. Self-reported symptoms of depression, anxiety, perceived stress and psychological inflexibility were collected before (T0) and after the intervention (T1). Qualitative data on the participants’ experiences of TARA were collected in focus-group interviews conducted halfway through the program and upon completion of the program. Individual interviews were also conducted 2 years later. Qualitative content analysis was performed.

**Results:**

The mean attendance rate was 61.22% and the dropout rate was 17.40%. The Child Session Rating Scale administered after every session reflected an overall acceptable content, mean total score 34.99 out of 40.00. Trends towards improvement were seen across all outcome measures, including the Hospital Anxiety and Depression Scale Anxiety (*t =* 1.13, *p* = 0.29) and Depression (*t* = 1.71, *p* = 0.11) subscales, Perceived Stress Scale (*t* = 0.67, *p* = 0.51) and Avoidance and Fusion Questionnaire for youth (*t* = 1.64, *p* = 0.10). None of the participants deteriorated markedly during the intervention. Qualitative content analysis resulted in a main theme labeled: *“An uncommon meeting-ground for personal empowerment”*, with 4 themes; *“Acknowledging unmet needs”*, *“Entering a free zone”*, *“Feeling connected to oneself and others”* and *“Expanding self-efficacy”.*

**Conclusion:**

TARA is feasible and acceptable in a mixed sample of Swedish medical students. The students’ reports of entering *an uncommon meeting-ground for personal empowerment* supports effectiveness studies of TARA in this context.

## Background

Medical students experience higher rates of depression, burnout, anxiety and suicidal ideation compared to the same age-group of the general population [1–3]. For example, large meta-analyses confirm the results that half of the medical students worldwide suffer from burnout even before residency [1]. Data from 129,123 medical students in 47 countries demonstrated that 27.2% of the students screened positive for depression and 11.1% reported suicidal ideation during medical school [2]. Yet another meta-analysis found even higher rates of depression and slightly lower rates of suicidal ideation [4]. The comparative prevalence varies, with medical students displaying between 2,2- and 5,2-times higher depression rating scores than individuals of similar age in the general population [2].

Matriculating medical students score better on indicators of mental health than matched college graduates [5], however, mental health deteriorates when entering medical school [2, 6–8], suggesting that medical school may in itself be a contributing factor [2]. Research addressing this matter has contributed to an increased awareness of the potential harmful effects of stress during medical school and residency, and many appropriate steps have already been taken to mitigate these [9, 10].

Mental disorders in medical students seem to be multifactorial in their origin [6, 11] and the effects of depression include well-established increased risk of suicide as well as a long-term risk of future recurrent depressive episodes [12]. Furthermore, depressive symptoms may adversely affect both study results and the long-term health of physicians [6], thereby reducing workplace performance, including quality of care and patient safety [13, 14]. Depression is a preventable and treatable disorder [15]. However, medical students are less likely than the general population to seek appropriate professional help, care or treatment and different mechanisms for this have been investigated [16–18]. Importantly, affected students show greater concerns about the stigma of mental illness compared to the general population [19], potentially explaining why less than 16% of students who screen positive for depression seek treatment [2]. Resident physicians across specialties seem to struggle to stay mentally healthy and further increases in depressive symptoms occur with the onset of residency training [20]. The suicide rates in male and female physicians are increased with 40 and 130% respectively compared to the general population [21], a trend starting already in medical school [22].

This data implies that appropriate and effective prevention methods have neither been developed nor successfully implemented. Rigorously designed prevention-studies specifically in medical students have been warranted, rather than “piecemeal” efforts to “treat” students’ and residents’ mental disorders [23]. Methods that aim to strengthen resilience, wellness and a sense of purpose and meaning, while also addressing the contextual factors and organizational structures are highlighted [23]. A 2020 Cochrane review concludes that with regards to resilience training for healthcare students “high quality replications and improved study designs are clearly needed” [24].

In an attempt to meet this need, we translated the manual for Training for Awareness Resilience and Action (TARA) into Swedish in close collaboration with the original author. We kept the core structure and content of the manual and adapted only slightly the psychoeducational presentations to better fit the more advanced pre-understanding expected in medical students. No other cultural adaptation was necessary. TARA is a novel, neuro-scientifically based program that was developed at University of California San Francisco [25]. In summary, the program is built on and arranged within the National Institute of Mental Health’s Research Domain Criteria (RDoC) framework [26] to address the major domains of function involved in adolescent depression, taking into account both fundamental neurobiological and developmental aspects of depression in this age-group.

TARA is a manual-based 12-week program, organized in a progressive manner that gives priority to the domains of function thought to be driving the depressive psychopathology in adolescents. TARA has shown feasibility in both adolescent clinical and non-clinical US populations and effectively targets symptoms of anxiety and depression in clinically depressed adolescents [27]. Data from mixed clinical and community samples indicate that postulated brain-changes are achieved in response to TARA [28, 29]. Since brain maturation continues into the third decade of life [30–33] we proposed that not only adolescents, but also young adults should be targeted with age-adapted prevention and treatment programs.

The aim of this single-arm pilot study was to test the feasibility and acceptability of TARA as an indicated prevention of mental disorders and stress-related symptoms in self-selected medical students. Feasibility and acceptability were evaluated with recruitment-rates, attendance, retention as well as session rating scores. Pre-post measures were used to evaluate feasibility with regards to symptom severity change. We also aimed to qualitatively explore the students’ experiences of participating in TARA both during and after the program, as well as at a two-year follow-up.

We used a mixed methods design to evaluate feasibility and acceptability as well as to explore the experiences of participating in TARA, including the subjective impact at a 2 year follow up. This trial was registered at clinicaltrials.gov on 28/09/2021, identifier: NCT05059392.

## Materials and methods

### Participants and recruitment

Participants were recruited from Umeå University School of Medicine. The University is in the north of Sweden and enrolls 130 medical students per semester. Participants were recruited from the second and fourth semester (year 1 and 2 respectively) through oral and written information at a mandatory class on professional development with a total attendance of approximately 225 students. Participation was voluntary and in addition to being a medical student there were no specific inclusion or exclusion criteria. Participants were allocated to one of two TARA-groups (A and B) based on their personal weekday preference, since the groups were held on different days of the week.

### Intervention format of TARA

The development of the TARA program was inspired by the structure and content of mindfulness-based approaches but is fundamentally different in several ways. First, breath and synchronized slow movements are used to improve emotional self-regulatory skills, rather than primarily focusing on acceptance of emotional experience through metacognition. There is a focus on “real world” relevance for the participants and a transgenerational dialogue and inquiry are emphasized. The explicit rationale for each practice is given, often with a scientific background explained in the psychoeducational presentations. Participants are encouraged to self-adjust their level of participation, to express their own emotional experiences and to validate other group members. Value-based committed action is an extended goal of the curriculum, not only equanimity and personal well-being. Time is spent contextualizing depressive symptoms and stress, investigating some of the negative impacts that culture and systems relevant to the participants have on their personal health.

The conceptual framework that informs the TARA intervention and a detailed description of the content of each module as well as each session has been thoroughly elaborated in a previous publication [25]. In summary, TARA contains psychoeducation and training of autonomic and emotional self-regulation by slow movement and breath-work, interoceptive and meta-cognitive awareness, relational and compassion skills, contextualization of symptoms and experiences, and finally community-based committed action. Thus, TARA first incorporates developmentally appropriate training to build emotion regulation skills, and, once these skills are acquired, practices including cognitive top-down control are introduced. The primary key target is to decrease amygdala hyperactivation, which is hypothesized to drive the pathophysiology of youth depression. Practices designed to increase vagal afference are therefore maintained during the whole 12-week period. Home practice of TARA-skills are encouraged and audio tracks of breathing- and movement exercises, as well as short, guided meditations are provided to all participants.

### Facilitator training and setup

Each session of the two respective TARA-groups was led by the same two facilitators, together covering expertise in contemplative practices and clinical psychology or psychiatry. The facilitators’ role during sessions was to teach specific content and to model a collaborative, inclusive, non-judgmental and supportive attitude. The senior author EH, who developed the TARA intervention, co-facilitated group A and observed all sessions of group B to provide ongoing supervision and training to facilitators and to monitor fidelity to the manual, both in terms of content adherence and the process of delivery. The facilitators were also videotaped to facilitate supervision and implementation of the protocol. Participants who did not show up for three consecutive sessions were contacted over the phone to discuss the reasons for not participating, and to assess their status and safety.

### Monitoring instruments

The Outcome Rating Scale (ORS) and Session Rating Scale (SRS) are 4-item self-assessments on 10-cm visual analog scales delivered on paper, higher scores indicating better functioning or experience [34]. Scores were manually transformed from visual analog scales to continuous scales, range 0–10 on each of the 4 items. Participants completed the ORS before each session, rating how they had been doing 1) individually, 2) in the family, 3) in school, and 4) overall, in the past week. The ORS was used as a safety measure to ensure that no student deteriorated severely in their mental health during the intervention and was not further analyzed. The SRS, a measure of working alliance, was completed after each session. SRS rates the session in terms of 1) how much they felt listened to, 2) how important the content and activities were to them, 3) how much they liked the session, and 4) their overall experience. The SRS mean score was used as an acceptability measure. Reliability and concurrent validity for both ORS and SRS are good [34].

### Other measures

Prior to the start of the intervention (T0), participants completed an online self-report questionnaire. The outcome measures were filled in again after the intervention, 3 months from baseline (T1). These included:

The Hospital Anxiety and Depression Scale [35], a 14-item self-rating instrument for anxiety and depression that is widely used [36, 37] in both somatic-, psychiatric- and primary care. Psychometric properties are good to excellent [38, 39]. Measured on a 4-point ordinal scale with total-scores ranging from zero to 21 on each of the 2 subscales measuring anxiety (HADS-A) and depression (HADS-D) respectively. Higher scores indicate more severe symptomatology. Example items are for anxiety “I get sudden feelings of panic” and for depression: “I still enjoy things I used to enjoy”. On both subscales using ≥8 as cut-off score for caseness is generally considered optimal, with few false negatives but a definite proportion of false positives [37, 40]. The factor-structure has been confirmed in a Swedish population sample where norm scores where 4.55 points (HADS-A) and 3.98 points (HADS-D) [41]. The factor-structure as well as the cut-off scores for caseness have been replicated in Scandinavian primary care [40]. In the present sample, the internal consistency was ***α*** = 0.85 (HADS-A) and ***α*** = 0.78 (HADS-D).

Perceived Stress Scale (PSS) is a ten-item measure of perceived stress in the last month. Measured on a five-point ordinal scale with possible total-scores ranging from zero to 40, higher scores indicate more severe stress. An example item is: “In the last month, how often have you felt that you could not cope with all the things that you had to do?” The scale has been validated in Swedish adults [42] and the internal consistency in the present sample was Cronbach’s ***α*** = 0.86.

Avoidance and Fusion Questionnaire for Youth (AFQ-Y) is an eight-item measure of psychological (in)flexibility rating: (a) cognitive fusion, (b) experiential avoidance, and (c) inaction or behavioral ineffectiveness in the presence of unwanted internal experiences [43]. Example item: “The bad things I think about myself must be true”. Measured on a five-point ordinal scale with possible total-scores ranging from 0 to 32, higher scores indicate less psychological flexibility. The scale has been validated in Swedish adolescents [44, 45]. The longer version AFQ-Y17 has successfully been used in studies of adult psychology students [46]. The internal consistency of the eight item AFQ-Y used in the present sample was Cronbach’s ***α*** = 0.75.

We also collected data on the same three questionnaires at a two-year follow-up, response rates were however too low to warrant their reporting (*n* = 10, 43.48%).

### Statistical methods

The dataset was checked for illogical values. For both categorical and continuous variables, responses outside the possible response categories were coded as missing. Descriptive statistics were calculated using standard measures. Sum scores, as well as means and standard deviations were calculated for each self-report measure. Missing data on the item level was imputed using Multiple Imputations (*n* = 1 missing item (0.60%) on AFQ-Y at T1) with 5 iterations, to enable calculation of sum score despite the missing item. The T1 scores on each item on HADS-A, HADS-D, PSS and AFQ-Y were used as predictors in the imputation model and we used a random seed. The results from imputed data did not differ from the results without imputation, imputed results are however reported to keep the sample intact for interpretative simplicity. The data was checked for normality using histograms and Q-Q plots, as well as skewness and kurtosis. Boxplots were used to check for outliers.

To evaluate potential differences between the test-retest sample and the baseline sample we used Fisher’s test for nominal data, Mann-Whitney U test for ordinal data and non-normally distributed continuous data and unpaired T-test for normally distributed continuous data. The same strategies were used to evaluate potential differences between participants in the two TARA-groups.

Paired samples T-tests were conducted to compare HADS-A, HADS-D, PSS and AFQ-Y scores at T0 and T1. All analyses were performed using SPSS statistics (version 26, IBM Corp., Armonk, NY, USA). All significance testing was two-tailed, with a significance level of 0.05. *P*-values were not corrected for multiple comparisons.

### Qualitative methods

A semi-structured focus-group interview about the participants’ experiences of TARA was conducted halfway through the program (after group B session 6, *n* = 8). Those who were present at the session and willing to stay afterwards were interviewed and the same participants were interviewed again upon completion of the program (after session 12, *n* = 4). Focus-group interviews were also conducted on individuals from group A, the technical quality of the recording was however too poor to include the data without compromising trustworthiness. The focus-group interviews were conducted by an independent researcher (JvK) without any involvement in the intervention and a semi-structured interview guide was used.

At the two-year follow-up another independent researcher (AB) conducted similar individual interviews with participants from both groups. These interviews were held online or at the interviewees’ choice of location. All participants were invited by email and six responded and agreed to participate. Four of them had attended group B and two had attended group A, three individuals had previously participated in at least one of the focus-group interviews. The semi-structured interview guide for the individual interviews was adapted for the current timeframe. Questions such as: “What are your experiences of participating in the TARA-program?” were reframed into: “what are your experiences of having participated in the TARA-program?”. Additionally, a few questions relating to the period between the end of TARA and the follow-up interview were added, such as: “Has there been any negative or undesirable outcomes since then that you relate to TARA?”. The questions were broad or open. Experiences before, during and after TARA were covered. Interviews lasted for 46 to 66 min, mean interview time 56 min (time of the audio-recorded interview excluding pre-interview information). Participants received no compensation for the interviews.

The interviews were audio-recorded, transcribed verbatim by the respective interviewer who were familiar with the data. The transcribed data was subjected to qualitative content analysis, which involves the systematic interpretation of the overt and underlying content and can be used to analyse participants’ reflections, experiences and attitudes [47–49]. The text was first read several times separately by the authors, discussed to get sense of it as a whole, and then divided into meaningful units relevant to the aim of the study. Material derived from answers to leading questions and nonsense utterances was discarded at this stage. The meaning units were then coded and codes were sorted into groups according to their variations, similarities and differences to create subthemes and more general themes. For example, codes such as *coming into a calm room, never regretted having every Wednesday scheduled*, and *good to have a place at home for it* were grouped together, abstracted, and interpreted to form the subtheme *Having time and a safe space* which was further abstracted and interpreted in the theme *Entering a free zone* under the main theme *An uncommon meeting-ground for personal empowerment*. The authors met regularly to discuss their interpretations and finally agreed on the structure of the themes.

## Results

### Quantitative results

A total of 23 students agreed to participate. Of them 10 were allocated to group A and 13 to group B. There were no significant differences between the individuals in the two groups on any of the studied variables. All results are presented with data from the two groups analyzed together. In total, 21 responded on the baseline questionnaire and 17 on the follow-up questionnaire. Sixteen individuals responded to both questionnaires (*n* = 7 from group A and *n* = 9 from group B).

#### Sample baseline characteristics

Sample baseline characteristics included age (mean 25.38, range 19–42, SD 5.49) and sex (5 males, 18 females, 1 participant with female sex reported non-binary gender). Eighteen participants (85.71%) were studying fulltime, two (9.52%) had individual study plans and one (4.76%) was studying part time and had part-time sick leave for burnout/depression. Six participants (28.57%) reported having a concurrent job besides fulltime studies. Seven (33.33%) reported having received medical care for mental health problems in the last year, both in specialist and in non-specialist settings (mean number of appointments was 5.67, range 2–10, SD 4.08). Ten participants (47.62%) reported economical stress and four (19.04%) reported feeling unsafe in their neighborhood. All were Swedish citizens and 12 (57.14%) identified themselves as being Swedish, three Finish, one Persian and one Swedish/Russian.

Baseline scores on the outcome measures in the whole sample were HADS-A (*n* = 21) (mean 10.71, range 3.00–21.00, SD 5.07), HADS-D (*n* = 21) (mean 5.62, range 0–14.00, SD 4.10), PSS (*n* = 20) (mean 23.25, range 13.00–36.00, SD 6.45), and AFQ-Y (*n* = 21) (pooled mean 14.97, range 3–25, pooled SD 6.14).

#### Qualitative sample descriptive statistics

In the focus-group interview sample from group B (*n* = 8) one was male and seven were female (mean age 23.29, range 20–29, SD 3,64). In the individual interview sample (*n* = 6) two had attended group A, four had attended group B and three of them had previously also participated in the focus-group interviews. One was male and five were female (mean age 22.50, range 19–30, SD 3.83). Baseline scores on the outcome measures in the whole qualitative sample (*n* = 11) were HADS-A (mean 10.80, range 4.00–19.00, SD 5.57), HADS-D (mean 4.30, range 0–14.00, SD 3.97), PSS (mean 21.30, range 13.00–35.00, SD 6.24), and AFQ-Y (mean 14.10, range 6–20, SD 5.26).

#### Attendance, retention and acceptability

In the total sample (*n* = 23) the mean number of attended sessions/participant was 7.35/12 (61.25%), range 2–11, SD = 2.59. The mean attendance rate per session was 14.08/23 (61.22%), range 8–18, SD = 3.42. Two (8.70%) participants attended only the first two sessions. Four participants (17.40%) attended less than half of the sessions and were classified as dropouts. The SRS, which was administered after every session, reflected an overall level of acceptance for the content; mean weekly individual total score was 34.99 out of maximum 40.00 (87.48%), range 26.83–39.80, SD = 4.17.

#### Other outcome measures

Data presented here are from the participants with both test and retest measures, *n* = 16. The test-retest sample was not significantly different from the baseline sample on any of the variables collected in this study. Results from paired samples T-tests are displayed in Table 1. The number of DF is not reported for the pooled analysis of AFQ-Y, as SPSS does not support its accurate approximation [50], the sample size is however still *n* = 16 and 5 iterations were used.

**Table 1:** Paired samples T-tests for change between T0 and T1

Variable	T0		T1					
	Mean	SD	Mean	SD	t (DF)	*p*	95% CI (change)	SD (change)
HADS-A	11.50	5.18	10.94	4.92	1.13 (15)	0.29	+ 0.50–1.63	2.00
HADS-D	6.00	3.98	4.81	3.23	1.71 (15)	0.11	+ 0.30–2.67	2.79
PSS	23.25	6.63	22.19	7.80	0.67 (15)	0.51	+ 2.33–4.45	6.36
AFQ-Y (pooled)	15.64	6.52	13.63	6.96	1.64	0.10	+ 0.39–4.42	4.93

*HADS-A* Hospital Anxiety and Depression Scale, Anxiety subscale, *HADS-D* Hospital Anxiety and Depression Scale, Depression subscale, *PSS* Perceived Stress Scale, *AFQ-Y* Avoidance and Fusion Questionnaire for Youth

### Qualitative results

The analysis resulted in 12 sub-themes that were sorted, interpreted, and abstracted into four themes and a main theme describing the participants experiences of participating in TARA (see Table 2). Extracts from the interviews were used to illustrate subthemes and support analytical claims, all quotes have been translated from Swedish to English [translator’s clarifications are bracketed].

**Table 2:** Overview of the qualitative results

Sub-theme	Theme	Main Theme
- Managing pervasive stress - Asking for customized teachings and being receptive - Creating conditions for well-being	Acknowledging unmet needs	An uncommon meeting-ground for personal empowerment.
- Having time and a safe space - Using one’s body, breath and attention to relax - Aiming to practice regularly	Entering a free zone	
- Developing new ways of relating to oneself - Being more authentic with others - Being part of a community	Feeling connected to oneself and others	
- Accessing useful strategies - Getting one’s priorities straight - Progressing from being led to leading oneself	Expanding self-efficacy	

#### An uncommon meeting-ground for personal empowerment

The findings indicated that the participants experienced TARA as “*An uncommon meeting-ground for personal empowerment*”. Our interpretation of the findings is that in TARA the participants experienced that unmet needs were acknowledged and that they entered a free zone. Furthermore, they experienced feeling connected to oneself and others and that they expanded their self-efficacy.

***Acknowledging unmet needs*** The potential of TARA as a means of acknowledging and addressing perceived needs in both themselves and others was central in the participants’ stories. This theme includes the participants’ accounts of managing pervasive stress, asking for customized teachings and being receptive, as well as creating conditions for well-being.

*Managing pervasive stress* The participants expressed that stress was present to some extent in their lives, some more than others. Many were under the impression that stress was a part of everyday life in society in general and even more so for medical students: “*I think that we are all very stressed out”* (IP2). Some expressed concerns about approaching burn-out.

*- If we can counteract mental ill-health, we will be better physicians. More students would make it the whole way because it feels like many get burned out on the way to becoming a physician. (IP5)*

Some participants saw a relationship between stress-management and being able to complete their medical studies, even to the point of suggesting ways to implement something like TARA in the core curricula of the medical and other university programs, possibly in mixed groups. Other participants did not see that as feasible logistically and advocated a more context-adapted version as an extracurricular course offered by the university.

It was reported by some participants that even to set time aside once a week to participate in TARA had induced stress. It was however commonly experienced that this kind of stress subsided as a result of doing the practice in the TARA-group.

*- To me it was a bit hard to go at first, because of being stressed over school and having many other things to do. So, at times it was a bit inconvenient to go, not because it was going to be difficult to be there, but actually getting there. But then I always left [TARA] just not understanding how I could have thought not to go. (IP6)*

The participants generally assumed that stress was going to continue to be present in their lives. To understand its causes and be able to manage it seemed to be a realistic outcome according to the participants. As a result of TARA, they reported an increased awareness of what stressors they had, as well as new skills to effectively manage them. Examples were given both from their own daily life and from helping others.

One student reported:

*- Like the studies for example, let’s say I studied all day and when I finish, I’m really finished. Even if there are things to do still. It can be there, I do not let it occupy my mind, instead I cook my food in peace and go to bed not thinking about it. That is something I didn’t really have before, that I do have now. (IP3)*

*Asking for customized teachings and being receptive* Participants described previous experiences of stress management programs and therapy, as well as body-oriented training, breathing exercises and meditation. It was reported that the theoretical underpinnings of the previous programs and therapy had left them unsatisfied and that they wanted a more solid theoretical framework for stress management practices. Regarding the theoretical components of TARA, their opinions differed. Many of them perceived the teaching content as being too basic for their own level of knowledge and recommended a more comprehensive theoretical framing and rationale, especially to convince sceptics of the program in the case of broader implementation. It was suggested by some to omit teachings in favor of exercises in the last two sessions, others found the teachings helpful and relevant throughout.

*- Some things were perhaps a bit basic; things I had heard before. Sure, repetition is ok, yet it was like nothing was added that was new. Nothing really stuck or made a wow-impression. (IP3)*

*- That [teaching component] was great, I think my favorite part of TARA. I remember that we talked about the limbic system in the brain among other things. And I think it was very interesting to be able to connect mindfulness to physiology, that was cool to know more about, I think. (IP6)*

As the participants recalled the time when they signed up for TARA both skepticism and curiosity was expressed. According to the participants, open-mindedness was perceived to be a prerequisite to develop an interest in the TARA-program as well as to receive and continue with the different TARA practices.

*- I tried to think of persons for whom TARA would not be suitable and I guess that would be the ones who are not mature enough to take it in yet, maybe too stressed and not willing to allocate the time. (IP2)*

As all participants reflected upon their own help-seeking behaviors both before and after TARA it was described that after TARA, they had noticed both an increased openness and disposition to ask for support when needed, and also a reduced need for help. Some participants reported that they had sought professional help for their mental ill-health at some point during the 2 years follow-up period. An increased ability to talk openly about difficult topics was reported as a result of TARA, yet some compared subsequent psychological treatment to TARA and were disappointed.

*- Afterwards, I have compared the things I do, like groups, to TARA and been disappointed, because I think TARA was better. (paus) It felt more evidence based. (IP2)*

*Creating conditions for well-being* The participants reported an improvement in their ability to create conditions for their own well-being, even outside of TARA. Participants with previous experience of working in healthcare reflected upon the usefulness of this ability: *“I know what it is like to work in healthcare, and I felt that this is really going to come in handy later.” (IP1)*.

The participants described a range of things that had contributed to the lack of well-being in their daily lives, including poor performance despite intense studies, having left their hometown and now living alone. While being used to challenging settings and time constraints, TARA was described as a less demanding environment with low-pressure to achieve. The group size and the practices done, as well as the general attitude conveyed was said to enable the release of unpleasant emotions and worry, as well as enable more positive experiences to emerge.

*- It was just a very open, nice environment. All were kind and listened to each other. I don’t know, I guess I just felt seen, heard, and safe. (IP6)*

*- Everything was just very unpretentious. It was just so nice when coming from school or anywhere else where you need to hold yourself, to come there where there are no demands, and you can do just as you wish. (IP1)*

It was also reported that even if one is feeling completely well to start with, to sign up for and participate in TARA can be fun and exciting. At the 2 year-follow up the participants’ descriptions ranged from being completely well to being on sick leave due to burnout. One participant who had been referred from primary care to specialist psychiatric care made the reflection that she might have been in that situation 2 years earlier if she had not participated in TARA.

***Entering a free zone*** The participants described experiences of entering a free zone in TARA. The contrast that this offered to their general descriptions of being busy and under pressure, mainly induced by themselves, was central in the participants’ stories. This theme includes the participants’ accounts of having time and a safe space, using one’s body, breath and attention to relax, as well as aiming to practice regularly.

*Having time and a safe space* The participants expressed that they appreciated the time and the arena that the weekly meetings offered. The scheduled sessions, while taking time from other things, was said to enable a pause that was positive and otherwise difficult to achieve in daily life.

*- To me it was a lot in the beginning, like a whole evening. But I thought it was good because that is the time you need to give it. It would not have been the same if it had been 1 h twice a week. It took maybe 45 min to even rev down. I think that was good in itself because then I started to prioritize my recovery more. Then I realized that perhaps you need more than 2 h of recovery each week. (IP2)*

The participants described that they entered a calm and safe place that they were not used to. In that place cell phones, schedules and other stressors were said to not affect them as much, even to the point where they became non-existent. One said: *“You get very calm, because you know what awaits you is one and a half hour where there is nothing else, other than that room” (IP3).* Some of the participants said that they tried to create a similar free zone at home and perceived this to be good for them. Others advocated to practice together and suggested that this could be done both in the morning and in the evening time.

*Using one’s body, breath and attention to relax* To move the body in new ways was uncomfortable for some of the participants in the beginning. However, all expressed feeling good about the movement as they got used to it. To actively use the body in this way was said to be helpful to increase focus for the participants who had difficulties with that. Some participants also experienced that attending to the breath was an effective way to let go of thoughts and become more present as well as more relaxed.

*- The breathing exercises were very good. Sometimes when trying to relax in the whole body and just lay still, to me that can be very stressful, it is very hard to keep the thoughts from spinning around way too much. To just breathe at that time, feels like you don’t have to think and yet you have something to focus on, the breath. So, the thoughts don’t go around, and you don’t feel more stressed doing them. (IP4)*

The participants described that they were neither used to observing the way they breathe, nor using their breath to self-regulate. Examples of how the participants had applied the different breathing techniques ranged from using them for increased physical performance and to calm down and anchor the attention in times of stress.

*- I can use the breath if I get stressed or so, or if working out with a strenuous exercise I can breathe in a better way to manage it. So basically, in everything. But I experienced that before TARA I was breathing just without knowing it, then afterwards I came to think about it now and then like ah, I can breathe in different ways. (IP6)*

The participants described that their previous attempts to relax had not always been effective and for some they had even induced paradoxical reactions of hyperactivation. One said: *“I have like an inner stress and feel like when you start to try to relax it is as if the pulse rises instead of slowing down, and you just get more stressed because of thinking of all the things you should do instead of relaxing” (IP4).* In TARA the participants described having felt into the body as a way to notice tension that subsequently could be softened. They also described that they enjoyed the relaxing body-scans and some reported difficulties in staying awake or even falling asleep at times during the sessions.

*- It was very nice to just attentively lay down and be here and now, just feel what it feels like in the body, if it was a guided meditation or whatever, also just very relaxing. Being here and now and let the thoughts come and go. (IP5)*

*- Body scan, that was actually one of the things that I used at home. I am very positive towards going through the body this way. Feeling the head, the face, the shoulders, relaxing in every joint. Very positive, it is rare that you relax the body in this way. (IP3)*

Instead of being still in times of anxiety some participants expressed a desire to move which was met in the movement practices of TARA. Others used the movement practice to wake up in the morning or to calm down and relax in the evening, they did not find it very helpful to move in times of acute stress.

*- During the breathing exercises I have quite a hard time staying focused on the breath, which I guess is kind of common, especially if you’re not used to it. But to do it in combination with movement has worked out really well for me and it is quick too. (focus-group interview)*

After some practice one participant described passing a threshold with the movements and she became more supple and able to fully breathe with the exercises. Previously, she reported a recurring pain in the chest upon taking deep breaths and further expressed an initial sense of stiffness in the whole body. The movements were described as non-revolutionary by more experienced participants; they were perceived as light and soft rather than a demanding physical exercise. A wish for more variation in the flow of movements was also expressed.

The participants further described that they had noticed how much time they spent thinking about other things than what they were currently doing. Some expressed that even during the sessions they were not always very present. As they learned how to focus their attention inside themselves, they were said to be less disturbed by the outside world and this they said, made the mind calmer. Regardless of where the participants came from, they reported an ability to calm down and be present here and now as a result of the sessions.

*- It is just like afterwards, no matter where you are coming from, you are more relaxed and feel more centered, as simple as that. (focus-group interview)*

The participants also described an increased presence in their bodies and in daily life, both being more aware of their physical state and current feelings, including in times of stress. In stressful situations they described that their changes in breathing pattern were more easily recognized and addressed. Some participants described this new kind of awareness as coming into contact with themselves again, others had re-established communication with their whole body.

*- In the beginning of TARA, I was unable to feel my toes, because of being so stressed. Then I practiced and now I am better at feeling into the body. (IP2)*

At the two-year follow up some participants still used a sequence of practices, to “de-stress” and fall asleep.

*Aiming to practice regularly* The participants’ descriptions of how much they used the exercises between sessions as well as after TARA ended varied and even in a given participant it was said to vary over time. Some expressed having learnt that it is possible to create new routines for oneself and some participants had continued to practice together, either self-lead or with recordings of the exercises. Others had tried inconsistently and some still discarded anything that they were not under pressure to do. Participants who studied remotely mentioned that to attend sessions was sometimes difficult logistically. Despite this and the challenging aspect to schedule time once a week, participation was said to have offered a bit of stability in daily life.

*- This with the time-aspect, having now scheduled every Wednesday for this semester, every Wednesday evening. I have never regretted that. It has given a bit of stability in daily life. Sure, we do have many scheduled things, but as I said before, I have this moment once a week and it has not brought anything negative. (focus-group interview)*

Some participants described a recurring resistance to dedicate a part of their time between sessions to do the TARA practices and for some the voluntary home-practice assignments were either not perceived as necessary, forgotten, or not prioritized. However, when taken up they were appreciated.

*- I thought they [the home assignments] were really good. I’m like if I get a task, I know that I am going to do it. But I liked that it was like you took a moment each day to do the assignments, then if you have a breathing exercise or a guided meditation, I take a moment for myself every day. And it feels very good to have that moment on your own, thinking and focusing on being here, then you become more relaxed, and it feels like you take the moment to just like, relax. I appreciated that a lot. (IP5)*

Some participants expressed that to sustain a home practice would have been beneficial for them. One said: *“It would have given me more if I had actually done the home-assignments, I’m sure of that” (IP6)*. Others suggested improved personal support to establish a daily practice to prevent future deterioration.

*- Today I’m not feeling all that well, I was on sick-leave for a month this summer, burnout-disorder. I go to therapy, ehm, started taking antidepressants a year ago. So, I would think that TARA kind of put a break on this negative spiral that I was in, and had I continued with something like it then I would maybe not have been in this situation today. (IP2)*

One suggestion that the participants expressed was to create a room on campus to meet around this practice during breaks, another was to continue with more sessions together. Participants also reflected upon the benefits and disadvantages of potentially making exercises mandatory instead of voluntary and concluded that somehow that would have defeated the purpose.

***Feeling connected to oneself and others*** The participants reported that overwhelming experiences at times had induced a sense of alienation for them and the re-establishment of lost connections through TARA was central in their stories. This theme includes the participants accounts of developing new ways of relating to oneself, being more authentic with others as well as being part of a community.

*Developing new ways of relating to oneself* According to the participants, TARA introduced a well-needed break from their commonly adapted performance-centered mode of being. Participants described that their previous relationships with themselves had involved high degrees of perfectionism, internal demands and critique, attitudes that were not always kept on a reasonable level. The self-image was said to have been very much dependent upon performing well, and this, together with a need for everything to be perfect for it to mean something, was said to make life stressful for them.

*- I realized I never knew I was being super ambitious. Or like, wanting to achieve a lot and things. (laughing) Not the best self-awareness at times. (IP1)*

*- And I was hoping that perhaps we would touch upon that a bit, or that I would adjust my attitude to it, because it can be quite rough to value yourself according to what you achieve of course. (focus-group interview)*

As a result of the practice, the participants reported being more calm and able to reflect more clearly. Through self-reflection they expressed noticing that the fact that they were empathetic towards others did not necessarily mean that they were at all empathetic towards themselves. They did however report an increased ability to be both empathic and kind towards themselves.

*- The biggest take-home I got was to adjust, I was going to say, to be kind towards myself and not feel like I must achieve a lot. I have taken that with me in basically most of what I do, like being late somewhere I can be really pissed at myself and then I just go why? I shouldn’t be, instead to be kind towards myself, like I would be to anybody else. (IP1)*

The participants reported thinking in new ways and expressed an increased acceptance for things as they are. Some mentioned not having to do certain things in a specific way anymore. Others were able to stay with unpleasant emotions and at times even enjoy the release of tears. Further, participants reported an ability to use the exercises to take a step aside from the chaos when feeling really bad, looking at oneself and the situation from a different perspective.

*- During the time of going to TARA I became a bit calmer, there was at least one evening each week when I could think a bit clearer, after TARA. (IP2)*

Some participants reported coming to terms with the approach of doing ones best, not judging the result and then moving on. One said: “*TARA has helped me to not take things so seriously, achievements and the like” (focus-group interview)*. Some said they had developed new hope that it will go well for them, both in their studies and more generally. This can be contrasted with the statement of one participant who expressed that before TARA she was feeling like a fraudster not deserving a space in medical school.

*- Some days perhaps I was having anxiety when going there, or generally. But I realized it got better. Perhaps it gave me some hope that it was fixable. (IP2)*

The participants furthermore stated that many people live their lives without deeply realizing what is important to them. They included themselves among those who at times ignore things while being on *“autopilot”* (IP6), as this seemed easier to do than to deal with reality. At the same time, this was also seen by them both as a risk factor for future mental ill-health and a cause to become more shut down and difficult to reach through voluntary programs such as the TARA-program.

*- Some people have, I wouldn’t say self-insight, seeing maybe not their deficiencies but perhaps areas of weakness or potential improvement, they will look for it, then there are those who have not yet realized that. They just go on and on and God forbid, hit the wall, or start feeling really bad and not know what to do. They are the ones I’m thinking about, how to reach them. (IP1)*

TARA was said to have opened up topics that were relevant to the participants and some reported new inspiration to identify their dreams and passions.

*Being more authentic with others* The participants expressed that to become more aware of what was going on inside of them was helpful not only to deal with challenging situations directly, but also to be more open about it with others which seemed to benefit relationships. An increased openness towards themselves was said to have increased their receptivity towards others too. The participants described an increased ability to be present with others and to take others’ perspectives. This was said to come easier through actively listening to what was said rather than think about other things, including what to say or do to solve the situation.

*- If you are not thinking about a whole lot of other things but can focus, when talking to someone you can focus more on that person and not let the thoughts carry you away. (focus-group interview)*

*- I would say it* [TARA] *has surely helped me. I think that if you learn to deal with what is going on inside of you, you can also be more aware of what you need. I think it benefits relationships if you have the tools, if being more aware of being stressed I can be more aware of how I act towards others and perhaps say: I am stressed out right now. Perhaps being a bit more open with how you feel. I think that it benefits, to become aware of how you are feeling and be open with it. (IP5)*

The increased awareness of the experiences of others was said to bring understanding and acceptance, which in turn was said to enable empathy to arise and be communicated. One participant who reported previous difficulties to even understand the situation of others, had learned that empathy was something that he could develop himself:

*- I always thought I was kind of, not un-empathetic but perhaps that’s what it is, when it’s hard to understand someone else’s situation. So, I think that the things we have talked about here and discussed has helped. Having experienced myself as not being very empathetic, I learned that that is something that perhaps can improve with training. (focus-group interview)*

It was described that the conversations during TARA were important and serious. One participant who had found it difficult to analyze herself in personal therapy described finding it helpful to *“see myself and recognize myself in others”* (IP4). The discussions with others were said to have revealed new ways of thinking and for some participants this reflective dialogue was more important than keeping the timing of the session. Some reported having improved communication with their partners as well.

*Being part of a community* An increasing sense of safety and belonging was expressed by the participants and an affinity with the group was developed that was said to weld them together. One said: *“It felt like we kind of melted together and became one”* (IP1)*.* As a result of TARA, the participants described having learned that they are not alone, in fact they expressed having more in common with the others than they initially thought. Previously they reported feelings of isolation from other students even in the same class, partly caused by studying a lot on their own. It was also common to feel inferior to other students that seemed to both know more and cope well, that contributed further to a sense of separation and insufficiency. The shared experiences in TARA were said to have strengthened the connections between them and give a sense of relief.

*- Many others are feeling the same way as I do, with stress and things. That you are not alone and even the people where you can’t really tell are experiencing it quite a lot. Many are stressed about different things in life, and it doesn’t always show. It gives me some relief to think that I’m not the only one. (IP4)*

The degree to which participants expressed a sense of community varied. Some did not feel they always gained much from the conversations and said that the break with snacks would have been enough for reflection, others mentioned that in their group there was not much of a discussion. Some participants reported that they still talked to each other and an increased ability to find friends with the right priorities after TARA was also reported. The participants described that identifying commonalities had been facilitated by the fact that they were all medical students. One said: *“We are all basically in the same boat, I could relate to others in a whole new way”* (IP1). Others expressed an openness to include students from other programs and non-students from other parts of society in future groups. TARA was further described as being different from group therapy. The group size was said to be large enough to be stimulating and small enough to allow for freedom of expression. Two participants explicitly mentioned the sense of community developed within TARA as being helpful.

***Expanding self-efficacy*** With regards to managing themselves and their own lives, the process of re-establishing an internal locus of control to expand self-efficacy was central in the participants’ stories of TARA. This theme includes the participants’ accounts of accessing useful strategies, getting one’s priorities straight, as well as progressing from being led to leading oneself.

*Accessing useful strategies* The participants mentioned that regardless of the degree to which a person has stress-related problems, there is always something to be found in TARA that can have a positive effect in your life. The participants expressed an appreciation for different TARA practices and the wide variety in the content was said to enable each one to pick and choose what worked best for them. One said: *“I took the pieces that suited me”* (IP2).

Most participants had signed up for TARA to learn how to better deal with difficult situations both in their studies and in life in general. Some participants described that they previously stood defenceless to inner and outer stressors, not knowing what to do when difficult things like anxiety came their way.

*- The tools, it is not like here is a solution to all your problems, you will have problems, your life is not going to be all milk and honey, it is just that you have the tools now. So instead of standing there helpless during a panic attack you can breathe like this, you can do like this, you can think like this, perhaps something will help you at least a bit. That is TARA. I feel like I have more tools to manage it now without these SSRI:s [antidepressants]. (IP1)*

Since even within a given person not all techniques were helpful at all times, participants described it as beneficial to have a range of different methods to calm down. Some participants mentioned that they had been given other and more practical tools from TARA compared to in previous individual psychotherapy. However, even if the participants described the tools as helpful, some also mentioned that they used them mostly when they had time and hence less of a need. Some said that it was not always easy to remember to access the tools one had found to be helpful.

*- Sometimes you forget to use it too, even if needed. It’s easy when you get stressed not to think very logically, like (name), now you can stop for a moment and breathe. Sometimes you are just all in it and you don’t think that far. I think you need a small reminder at times, how to deal with stress or like, that I know what to do it, it’s just good if I actually use the tools. Because I think it is really good when I do, and it works. (IP5)*

*Getting one’s priorities straight* The participants reported that as a result of TARA they reflected more on the reasons they had for doing the things that they would normally do. Medical school-related tasks were said to be given highest priority, knowing what it takes to achieve good results. However, stress was said to make it more difficult to stay with their own priorities. They found it particularly challenging to focus on and complete tasks with delayed reward in times of stress, for example studying or working out. Instead, they described finding themselves doing things that gave more instant reward yet distracted them from their commitment, and this was said to not always be very helpful or constructive. One said: *“Often when I get stressed, I start doing other things, remembering I have to clean, wash clothes, cook food or something that really isn’t all that important but feels important in that moment”* (IP4).

Compared to schoolwork, TARA-exercises were generally said to come second. The sessions were however said to be important, and some said they missed them only when they were sick. One said: *“Coming to TARA, that was my moment. Otherwise, it feels like school is top priority at all times”* (IP1).

*- Perhaps I didn’t get the time to prepare something for Thursday morning if we met on Wednesdays. I was quite ok with that in the end, to me it was more important to go to TARA. (IP2)*

For some participants the body seemed to take charge and prioritize TARA even when there was no active intentionality involved.

*- It was like my body was doing it the other way around. It is like when you say now, I should do the dishes, and your body is in the couch saying: nope, no energy. Instead, it was the other way around. I was telling myself it is ok, you don’t have to, and suddenly I just put the clothes on and just went out [to go to TARA]. It was like my body needs this, I am going to feel so much better both during and afterwards. (IP1)*

One participant had continued to find it difficult to prioritize her own health and expressed that because of that she was now awaiting admittance to specialist psychiatric care. Another described some situations in which she had become better at setting her intention and staying with it, as well as the positive effects of doing that.

*- I may be able to prioritize working out in a different way. Before I thought I should work out but had a lot of other things to do, so it was left a bit to the side. Now that pause of doing something else that reduces the stress, that improves the results of doing the rest. (IP4)*

*Progressing from being led to leading oneself* The participants expressed initial expectations upon starting TARA that someone else was going to solve their problems of not feeling well and some had not expected to do much themselves. On that account the participants expressed that starting with small sequences of facilitator-led training enabled them to begin to do exercises that would have been difficult for them to do without being led. One said: *“I remember that we had mindfulness and yoga, starting off gently, and then we did it more and more on our own, in our own pace”* (IP2). With time it was said that the growing understanding that they themselves had the key, enabled an increased sense of responsibility for their own situation.

*- The difference was that with the psychologist it was just me talking, I got questions and then I went on about everything and thought that she was going to help me tease out what the problem was and what I could do to get happy again. But it was not like that in TARA. (IP6)*

According to the participants their own abilities improved with training. They reported that TARA had given a foundation for stress reduction that they themselves could further build upon. Some participants said that they had modified the exercises according to what worked for them and some even developed new strategies on their own.

The participants described large and small victories along the path and examples of successful self-regulation in critical moments in their studies were reported.

*- When I was going to write this final exam that was to determine if I could stay or not, I sat down and looked through the questions and was just like: this is not going to work out, I’m not going to make it. Ah, the tunnel vision, I got warm and cold and I just ok. Put it down, went to the toilet, rinsed my face in cold water and sat down to breath and pat the body. Just be kind to yourself, it’s ok, it’s not the whole world and you’re not going to die, you know, well, TARA-stuff. I noticed how quickly it went away. Then I went back to sit down and could see it in a different way ... Then I wrote. After a while it came back, and I did the same thing again. It was huge for me to be able to ward off that situation, it was like I won somehow, and I know that was thanks to TARA. (IP1)*

## Discussion

This single-arm pilot study has shown feasibility and acceptability of TARA for medical students with the aim to prevent mental ill-health and increase their resilience. Feasibility and acceptability were evaluated with recruitment-rates, attendance, retention as well as session rating scores. Pre-post measures were used to evaluate feasibility with regards to symptom severity change. The recruitment rate (23/225, 10.2%) was acceptable and attendance (61.22%) as well as retention (82.60%) were good. Session Rating Scale-scores indicated that the participants were generally satisfied with the content. All the outcome-measures trended towards improvement and none of the individual participants deteriorated markedly during the intervention period. The participants experienced their participation in TARA as entering *an uncommon meeting-ground for personal empowerment*.

### Positive trends

Compared to medical students globally the anxiety-prevalence estimates in the participants in this study were higher. In total 66.67% screened above cut-off for caseness on the HADS-A subscale at T0. This is compared to a prevalence of 33.8% globally, which is also substantially higher than in the general population [3]. For depression 23.81% screened above cut-off on the HADS-D subscale, compared to prevalence rates of both depression and depressive symptoms in medical students of 27.2% [2]. The differences seen between our sample and the average medical student population may be due to the use of different questionnaires and cut-offs, affecting sensitivity as well as specificity. The small sample size reported here also induces a level of uncertainty. Importantly, it could mean that students with greater symptom severity show a greater interest in TARA as a potential means to increase their well-being. This was shown in a similar intervention study in which the self-selected students had greater baseline stress and anxiety scores than their control-group peers [51].

Both HADS-A, HADS-D, PSS and AFQ-Y scores trended towards an improvement post compared to pre-intervention. However, the group level effects were small, and the clinical relevance must be considered uncertain even had they been statistically significant. This was expected given the mainly non-clinical sample and the preventive approach of the study. Potential effects in less than healthy samples cannot be determined from this study. However, all students scoring above cutoff on HADS-D at T0 improved their scores at T1 which may indicate that there could be benefits gained from identifying high-risk groups among medical students for indicated preventive interventions. However, the results could also partially be explained by regression to the mean [52] and subgroup-analyses are not reported due to the small sample-sizes and risk of bias [53]. Among students scoring above cutoff on HADS-A at T0, there were 4 that scored higher at T1. No one deteriorated more than 3 points on either HADS-A or HADS-D, which we interpret as TARA being safe to apply in this context. The most significant deteriorations seen on the other scales were + 4 points (AFQ-Y) and + 11 points (PSS), possibly reflecting upcoming exams at the time of T1-assessments.

### Medical student empowerment

This study revealed that the medical students experienced TARA as an *uncommon meeting-ground for personal empowerment*, with experiences of both meeting others and themselves in new ways. The concept of empowerment has previously been studied mainly in diverse disadvantaged populations, see for example [54–57], and here we argue that the approach is applicable and valid also for individuals with high levels of functioning and with the privilege that being a medical student entails. Indeed, nursing education and practice has embraced the concept and identified a range of potential benefits, see for example [58–60]. In medical students on the other hand this line of research is strikingly lacking and limited to the inclusion of students in curricular development [61]. Recently there has been a suggestion to introduce developmentally appropriate empowerment strategies for residents, as this may lead to reductions in burnout incidence [62]. We propose to include medical students in this discussion, as intervening at an early stage is expected to have better preventive potential. The hypothesis that TARA increases a sense of empowerment in medical students remains to be proven in a randomized controlled design and our findings encourage such quantitative evaluations in future studies. If this hypothesis is proven TARA may increase resilience to common mental disorders [59, 62] as well as more widely affect the future integrity, sense of responsibility as well as sustainable leadership of the medical students of today. This may even have positive implications for future professional lives as physicians and benefit their future patients [62].

The mainly positive descriptions contained in the four themes identified, are interpreted as additional indications of TARA being feasible and acceptable. The participants’ descriptions also suggest that the students who self-selected for the intervention found this type of training worthwhile, which is in line with other qualitative studies of stress management programs for medical students [63, 64]. The participants main suggestion for improvement was to strengthen the theoretical framing and rationale in the psychoeducational presentations. We interpret this as a valid request for a more comprehensive empirical foundation, aligned with the preexisting knowledge base of medical students rather than a level adapted for depressed adolescents for whom the manual was originally developed. We will continue to update the material to further optimize the delivery to better fit medical students.

### Limitations and methodological discussion

This study is limited by its small sample size and the lack of control condition. Even though the CONSORT statement recommends that formal hypothesis-testing for effectiveness is not conducted in pilot and feasibility trials [65] we still reported all outcome measures for illustrative purposes here. The small effects were expected given the mainly healthy sample, furthermore, T1-assessments were collected at the end of the year, shortly before large exams, a time when medical students experience a greater level of anxiety and stress [66, 67]. While being an aspect of the naturalistic design of the study this may bias outcome measures to the disadvantage of TARA and may have contributed to the loss of follow-up. We did collect quantitative data at the time of the interviews 2 years later, response rates were however too low to warrant their reporting (*n* = 10, 43.48%), indicating that in future studies the long-term outcome data collection strategy needs to be improved for follow-up data-collection to be feasible and meaningful.

Self-selection of participants limits generalizability of quantitative findings to those with an openness towards and an interest in this kind of intervention and on the other hand it is possible that self-selected participants are just the right target-population for voluntary programs such as TARA. Some participants missed assessments altogether or were lost to follow-up and there is a possibility that non-responders were not satisfied with the program, biasing the results in favor of TARA. The SRS-results however indicate that this was not the case.

Aspects of feasibility that have not been evaluated include feasibility of recruiting participants in the presence of randomization to either TARA or control condition(s), feasibility of large-scale facilitator-recruitment and feasibility with regards to comprehensive evaluation of harmful effects.

Regarding credibility we consider it a methodological strength that the interviews were conducted by independent researchers that subsequently were involved in the analysis together with researchers of different age, sex and background/interpretative repertoires. The iterative process of repeated discussions between the authors during the analytic process, as well as the interviewers confirming the results, increased credibility and dependability, as well as confirmability. A benefit of our longitudinal approach to interviews was that longer-term impact and distant effects were possible to assess, even though some students were not sure themselves of what was a result of TARA and what was a result of other events that had occurred during the two-year period. There is an obvious risk that self-selection of participants for qualitative interviews compromises the range of experiences expressed, however, although the opinions expressed here were mainly positive, there were also clearly negative ones. This indicates that the range of experiences expressed was not compromised and validates our use of qualitative content analysis which is a method that emphasizes variation in data [47]. To conduct interviews both in groups and individually, as well as both online and in person can be considered a case of environmental triangulation, strengthening credibility further [68].

The main limitations to our qualitative approach include 1.) Having group-level interview data only from TARA-group B. Also, only two of the individual interviews were conducted with participants from TARA-group A, owing to participants not accepting invitations to interview. 2.) Interview data was not collected specifically from individuals who dropped out early from the intervention, an approach that might have added important information. Again, we were limited by acceptance-rates as invitations were sent out to all participants. 3.) Females were overrepresented in the qualitative sample. Therefore, since the degree to which a population is accurately represented in the sample determines sampling validity [69], increased sample variability would have potentially improved the analysis.

Another potential limitation is that we did not check our abstractions and interpretations with the participants themselves, even though the results were agreed upon by all the members of the research-team. The technique of performing member-checks with study participants is however controversial and not generally recommended in guidelines, see e.g. [70].

The author team involved persons both with and without personal involvement in TARA as well as in the university teaching environment at large. Entering the project from different angles uniquely enabled a mutual illumination of blind spots, it also facilitated us going beyond both simple replication of existing pre-understandings and superficial inquiry. Therefore, as we present these understandings and interpretations, we hope that the results will be more than narrowly context specific.

### Future directions

Results from the interviews indicated that TARA provided a wider range of benefits than those initially assessed with self-rating. Thus, TARA may have meaningful effects beyond those captured with current self-rating-scales, reinforcing the importance of qualitative assessment. In the future it will be critical to evaluate these aspects quantitatively for any firm conclusions to be drawn.

Students reflected upon some of the same issues as medical educators do, such as whether wellness training should be integrated into required curriculum for all students [71], and we propose that universities offer TARA as a voluntary safe option for self-selected students with perceived needs, potentially through the student health services.

As a future direction we recommend to further adapt the theoretical content of TARA to better match the pre-understanding of medical students. To conduct a randomized controlled trial with larger sample size and long-term follow up is the logical next step to test the effectiveness of the intervention, preferably including medical school-related outcomes like retention and study-results in addition to mental health measures. With a manual in English and data indicating that online delivery is equally feasible (unpublished data), to implement TARA for medical students more broadly could be easily achieved online. As an outreach effort this could potentially have the benefit of reaching more students, particularly in northern Sweden where the clinical training sites are dispersed over large geographical areas.

## Conclusions

TARA is feasible and acceptable in a mixed sample of Swedish medical students. Quantitative data showed positive trends and the students described having entered *an uncommon meeting-ground for personal empowerment*.

## Data Availability

The datasets generated and analyzed during the current study are not publicly available due to restrictions posed by the ethical review authorities on information that could compromise research participant privacy. They are however available from the corresponding author on reasonable request.

## Abbreviations

AFQ-Y: Avoidance and Fusion Questionnaire for Youth
ORS: Outcome Rating Scale
SRS: Session Rating Scale
HADS-A: Hospital Anxiety and Depression Scale – Anxiety subscale
HADS-D: Hospital Anxiety and Depression Scale – Depression subscale
PSS: Perceived Stress Scale
RDoC: Research Domain Criteria
TARA: Training for Awareness Resilience and Action
